# The Duration of Stress Determines Sex Specificities in the Vulnerability to Depression and in the Morphologic Remodeling of Neurons and Microglia

**DOI:** 10.3389/fnbeh.2022.834821

**Published:** 2022-03-07

**Authors:** Rita Gaspar, Carina Soares-Cunha, Ana Verónica Domingues, Bárbara Coimbra, Filipa I. Baptista, Luísa Pinto, António F. Ambrósio, Ana João Rodrigues, Catarina A. Gomes

**Affiliations:** ^1^Coimbra Institute for Clinical and Biomedical Research (iCBR), Faculty of Medicine, University of Coimbra, Coimbra, Portugal; ^2^Centre for Innovative Biomedicine and Biotechnology (CIBB), University of Coimbra, Coimbra, Portugal; ^3^Clinical Academic Center of Coimbra (CACC), Coimbra, Portugal; ^4^Life and Health Sciences Research Institute (ICVS), School of Medicine, University of Minho, Braga, Portugal; ^5^ICVS/3B’s –PT Government Associate Laboratory, Braga/Guimarães, Portugal; ^6^Faculty of Pharmacy, University of Coimbra, Coimbra, Portugal

**Keywords:** chronic stress, microglia morphology, sex differences, dorsal hippocampus, *nucleus accumbens*, neurons morphology

## Abstract

Stress exposure has been shown to induce a variety of molecular and functional alterations associated with anxiety and depression. Some studies suggest that microglia, the immune cells of the brain, play a significant role in determining neuronal and behavioral responses to chronic stress and also contribute to the development of stress-related psychopathologies. However, little is known about the impact of the duration of stress exposure upon microglia and neurons morphology, particularly considering sex differences. This issue deserves particular investigation, considering that the process of morphologic remodeling of neurons and microglia is usually accompanied by functional changes with behavioral expression. Here, we examine the effects of short and long unpredictable chronic mild stress (uCMS) protocols on behavior, evaluating in parallel microglia and neurons morphology in the dorsal hippocampus (dHIP) and in the *nucleus accumbens* (NAc), two brain regions involved in the etiology of depression. We report that long-term uCMS induced more behavioral alterations in males, which present anxiety and depression-like phenotypes (anhedonia and helplessness behavior), while females only display anxiety-like behavior. After short-term uCMS, both sexes presented anxiety-like behavior. Microglia cells undergo a process of morphologic adaptation to short-term uCMS, dependent on sex, in the NAc: we observed a hypertrophy in males and an atrophy in females, transient effects that do not persist after long-term uCMS. In the dHIP, the morphologic adaptation of microglia is only observed in females (hypertrophy) and after the protocol of long uCMS. Interestingly, males are more vulnerable to neuronal morphological alterations in a region-specific manner: dendritic atrophy in granule neurons of the dHIP and hypertrophy in the medium spiny neurons of the NAc, both after short- or long-term uCMS. The morphology of neurons in these brain regions were not affected in females. These findings raise the possibility that, by differentially affecting neurons and microglia in dHIP and NAc, chronic stress may contribute for differences in the clinical presentation of stress-related disorders under the control of sex-specific mechanisms.

## Introduction

Exposure to stress has a detrimental impact on certain brain functions, depending on the duration, type, and severity of stress. Uncontrollable stress is a contributing factor for major depressive disorder (Iwata et al., 2013), a severe and debilitating psychiatric illness characterized by a significant change in mood and accompanied by symptoms such as anhedonia and disrupted sleeping, eating, and cognitive deficits (Kessler, 2012).

A wide variety of animal models have been used to mimic human depression, but, as a heterogeneous disorder, many of its symptoms (depressed mood, feelings of worthlessness, and suicidal ideation) are hard to be mimicked in laboratory animals and, for an animal model to be valid, it is not necessary to exhibit all the traits of depression, since patients do not manifest every symptoms of the disease (Belzung and Lemoine, 2011).

Unpredictable chronic mild stress (uCMS) protocol is widely used and involves a permanent exposure to a variety of mild stressors in an unpredictable manner. In adult rodents, uCMS is a valid model of depression (Willner, 2005) and induce a variety of behavioral alterations, including anxiety, anhedonia, decreased exploratory behavior, and increased immobility/despair behavior when exposed to stressful environments, as well as impaired spatial cognition (Henningsen et al., 2009; Hill et al., 2012; Bessa et al., 2013; Morais et al., 2014; Patricio et al., 2015).

Stress impact various aspects of immunity that in turn promote stress susceptibility. As innate immune cells of the brain, microglia play an integrative role in maintaining neuronal homeostasis (Salter and Stevens, 2017). These cells are distributed throughout the brain and function as a critical line of defense against injury and pathogenic insults (Hanisch and Kettenmann, 2007). It has been reported that stress induces morphologic changes of microglia (Sugama et al., 2007), namely promoting microglial hyper-ramification in the prefrontal cortex (PFC) (Tynan et al., 2013), which supports the theory that these cells play an important role in modulating stress responses (Reus et al., 2015). In the healthy adult central nervous system (CNS), microglia have a ramified morphology characterized by long and thin processes that support the ability for searching potential threats for local homeostasis (Nimmerjahn et al., 2005; Kettenmann et al., 2011; Xavier et al., 2014; Wu et al., 2015). Some studies have described that when microglia respond to insults, they change their morphology, the processes retract and the cell body enlarges, giving microglia an amoeboid shape (Davalos et al., 2005; Cho et al., 2006). However, in our recent studies we report a diversity of morphologic changes that globally depend on the time of stress exposure (prenatal versus adult stress), on the sex of the animal and on the brain region under study (Caetano et al., 2017; Duarte et al., 2019; Gaspar et al., 2021). Our observations suggest that microglia remodeling upon stress are not limited to the acquisition of an amoeboid phenotype, as previously described (Sugama et al., 2007; Tynan et al., 2010; Kreisel et al., 2014), but instead may vary from different degrees of atrophy to hypertrophy.

In addition to microglial changes, several studies also point toward stress-induced sex differences in neurons morphology (Galea et al., 1997; Garrett and Wellman, 2009; Bock et al., 2011; Breach et al., 2019), although the majority of studies were performed exclusively in males (Magarinos and McEwen, 1995; Lambert et al., 1998; Radley et al., 2006; Bessa et al., 2009a, 2013; Morais et al., 2014; Melo et al., 2015; Patricio et al., 2015). In fact, stress-induced morphologic changes in microglia and neurons are associated with behavioral alterations in rodent models, including anhedonia, anxiety-like behavior and despair-like behavior (Fonken et al., 2018; Liu et al., 2019).

Sexual dimorphism at multiple levels, including cellular, molecular, and immune system in stress response suggest that stress-elicited neuroinflammatory priming may vary between sexes (Couch et al., 2013; Kreisel et al., 2014; Bekhbat and Neigh, 2018; Wohleb et al., 2018). However, little is known about the morphologic adaptation of brain cells in its relation with depression vulnerability between sexes when subjected to stress protocols of different duration. Therefore, in this study, we examined the effects of the exposure to short and long uCMS in both sexes upon behavior and plastic changes of microglia and neurons. We used a set of different behavioral tests to evaluate anxiety- and depression-like profiles of adult rats exposed to uCMS. Using an automated methodology, we quantified how uCMS alters several morphologic properties of microglia and neurons in the dorsal hippocampus (dHIP) and *nucleus accumbens* (NAc), two key brain regions in stress responses.

## Materials and Methods

**FIGURE 1:**
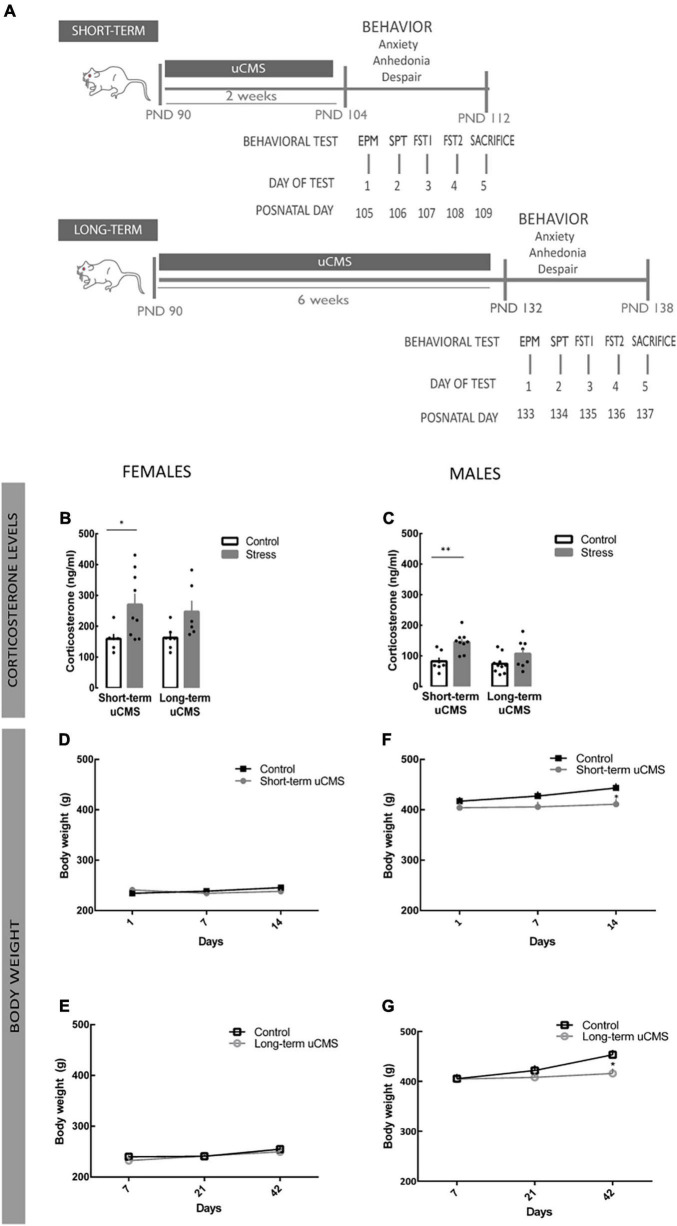
Unpredictable chronic mild stress (uCMS) induces a dysregulation of body weight in males and in the circadian corticosterone secretion pattern in both sexes. **(A)** Schematic drawing of the uCMS protocol. **(B,C)** Corticosterone serum levels measured at 8:00 a.m. in female and male rats exposed to stress in adulthood. **(D,E)** Body weight of female rats exposed to short- or a long-term protocol of chronic mild stress for 2 and 6 weeks, respectively. **(F,G)** Body weight of male rats exposed to short- or a long-term protocol of chronic mild stress for 2 and 6 weeks, respectively. Results are presented as the mean ± SEM of 10–20 animals (body weight) 6–10 animals (corticosterone); comparing with control, calculated using a two-way Analysis of Variance (ANOVA) followed by a Bonferroni *post-hoc* test. *p < 0.05 and ^**^p < 0.01.

The timeline of all procedures is shown in Figure 1A.

### Animals

Adult male and female rats (Wistar Han), 3-months old (Charles River Laboratories, L’Arbresle, France) were housed and kept under standard laboratory conditions: 22°C, 55% relative humidity, and 12 h light/dark cycle with free access to food and water. A complete timeline of all manipulations and behavioral tests is provided in Figure 1A. The handling and health monitoring were performed according to federation of european laboratory animal science associations (FELASA) guidelines. All experimental procedures were approved by european union (EU) – Directive 2010/63/EU and the Portuguese National Authority for animal experimentation, Direção-Geral de Animal e Veterinária (DGAV). All protocols were approved by the Ethics Committee of the Life and Health Sciences Research Institute and by DGAV (#19074).

### Unpredictable Chronic Mild Stress

At posnatal day (PND)90, animals were randomly divided into four experimental groups and placed in separate rooms: a group of animals exposed to uCMS for 2 weeks (Short-term uCMS – Stress); a group not exposed to uCMS for 2 weeks (Short-term uCMS – Control); a group of animals exposed to uCMS for 6 weeks (Long-term uCMS – Stress); a group not exposed to uCMS for 6 weeks (Long-term uCMS – Control). An adapted version of the previously described and validated uCMS protocol (Willner, 2005; Alves et al., 2017), was applied for two periods of different duration (2 and 6 weeks). The uCMS protocol consisted of a variety of unpredictable mild stressors, including confinement to a restricted space for 1 h, placement in a tilted cage (30°) for 3 h, housing on damp bedding, 15 h of food deprivation followed by exposure to inaccessible food for 1 h, water deprivation for 15 h followed by exposure to an empty bottle for 1 h, exposure to stroboscopic lights during 4 h and reversed light/dark cycle for 48 h, every 7 days. Rats subjected to stress were randomly exposed to 2–4 stressors every day for 2 or 6 weeks (Supplementary Figure 1). The controls were left undisturbed under the previously described maintenance conditions. Body weight was monitored weekly to monitor the overall effects of the stress paradigms.

### Behavioral Analysis

At the end of the uCMS protocol, a series of behavioral tests were performed in sequence to evaluate anxiety and depressive-like behavior. The Elevated Plus Maze (EPM) and Forced Swimming Tests (FST) were conducted during the light period of animals (9:00 a.m.–5:00 p.m.); the Sucrose Preference Test (SPT) test was performed during the dark period, from 9:00 p.m. to 10:00 p.m.

### Elevated Plus Maze

To assess anxiety-like behavior, the EPM test was performed at PND105 (short-term) and at PND133 (long-term). The maze (ENV-560; Med Associates Inc., St. Albans, VT, United States) has two closed (50.8 cm × 10.2 cm × 40.6 cm) and two open arms (50.8 cm × 10.2 cm), raised 72.4 cm above the floor and illuminated by a dim light. Each animal was positioned in the center of this elevated plus-shaped platform for 5 min. The performance of rats in EPM was video-recorded and subsequently analyzed. The ratio of time spent in the open arms *per* total time spent in the open and in close arms was calculated as an index of anxiety-like behavior.

### Sucrose Preference Test

This test was performed at PND106 (short-term) and PND134 (long-term). Briefly, after 12 h of food and water deprivation, rats were presented with two pre-weighted bottles containing tap water or a solution of sucrose 2% for 1 h. The liquid intake from each bottle was calculated by comparing the differences in bottle weights before and after the test. The sucrose preference was determined as the percentage of sucrose solution intake that was calculated according to the formula: SP = [sucrose intake/(sucrose intake + water intake)] × 100, as previously described (Bekris et al., 2005). Low sucrose preference represented anhedonia, a core symptom of depression. When the preference test ended, rats were given free access to water and food.

### Forced Swimming Test

The test was performed at PND107-108 (short-term) and PND135-136 (long-term) after SPT. On the 1st day, rats were placed individually in a glass cylinder with water (62 cm height; 25.4 cm diameter; depth no less than 50 cm, 23°C) for 5 min. Then, the rats were dried and transported back to their home cages. In the 2nd day, the rats were subjected to one 5-min session of swimming. The test session was video-recorded, and the immobility time of each rat was measured using the EthoVision XT 11.5 tracking system (Noldus Information Tecnhology, Wageningen, The Netherlands). Immobility was defined as floating state in the water, without struggling and making only those movements to keep the head above water. Depressive-like behavior was defined as an increase in the immobility time.

### Immunohistochemistry and 3D Morphometric Analysis of Microglia

After completion of stress protocols and behavioral tests, all groups of rats were deeply anesthetized with sodium pentobarbital (20%; Eutasil ^®^, Sanofi, Gentilly, France) and transcardially perfused with 0.9% saline. The brains were removed and one hemisphere from each brain was used for Golgi staining technique and the other for immunohistochemistry for ionized calcium-binding adaptor protein-1 (Iba-1) followed by the 3D reconstruction of microglia cells. The right hemispheres, used for Iba-1 immunohistochemistry, were post-fixed in 4% paraformaldehyde (PFA), cryoprotected in 30% sucrose overnight, and then embedded in Optimal Cutting Temperature compound (OCT, ThermoScientific, Waltham, MA, United States), snap-frozen and stored at −80°C. Coronal sections (50 μm) of the hippocampal dentate gyrus (DG) (stereotaxic coordinates of interaural 5.20 mm and bregma −3.80 mm) and NAc (stereotaxic coordinates of interaural 10.20 mm and bregma 1.2 mm) were further stained to visualize microglia cells. Microglia were visualized using the following protocol: free-floating sections were blocked 2 h with 5% bovine serum albumin (BSA) in phosphate-buffered saline (PBS) + 0.1% Triton X at room temperature (RT) and incubated for 48 h at 4°C with an antibody specific to Iba-1 (1:1,000; Wako Chemicals Inc., Richmond, VA, United States) in 5% BSA/0.1% Triton X/PBS. Iba-1 is constitutively expressed in microglia, being involved in cytoskeletal reorganization, and is up-regulated in response to microglial cell activation. Sections were then rinsed and incubated for 2 h at RT with the appropriate secondary antibody (donkey anti-rabbit, 1:1,000, Invitrogen, Waltham, MA, United States) and 4’,6-diamidino-2-phenylindole (DAPI, 1:5,000). Sections were rinsed and mounted on gelatinized slices, using glycergel (DAKO mounting medium, Santa Clara, CA, United States). Images of 10 random microglial cells from each animal were acquired with a laser scanning confocal microscope LSM 710 META connected to ZEN Black software (Zeiss Microscopy, Oberkochen, Germany) using a 63x objective lens (oil immersed, Plan-Apochromat 63x/1.40 Oil DIC M27). Microglia cells were reconstructed using the Neurolucida software (MBF Bioscience, Williston, VT, United States). Morphometric data related to branch analysis were extracted by the Neurolucida Explorer software (MBF Bioscience, Williston, VT, United States). The parameters analyzed were the total number and the length of cellular processes and their measures *per* branch order, considering processes of order 1 those emerging directly from the cell body, processes of order 2 those arising from processes of order 1, and so forth (Caetano et al., 2017).

### Neuronal Morphology

To assess the dendritic morphology of granule neurons of DG and spiny medium neurons of NAc, three-dimensional morphological analysis was performed on Golgi-Cox stained material. The left hemispheres were immersed in a Golgi-Cox solution (1:1 solution of 5% potassium dichromate and 5% mercuric chloride diluted 4:10 with 5% potassium chromate) for 14 days, cryoprotected with 30% sucrose solution for 72 h, and sectioned at 200 μm in a vibratome in a 6% sucrose solution. Brain sections were mounted on gelatin-coated slides, lightly pressed and kept in moist container until developed, clarified, and then cover slipped. For each selected neuron, dendritic branches were reconstructed at 1,000× (oil) magnification, using a motorized microscope (Axioplan 2; Carl Zeiss, Oberkochen, Germany) and Neurolucida Neuron Tracing Software (MBF Bioscience, Williston, VT, United States). For each animal, approximately 10 neurons were analyzed in the dHIP and in the NAc. Data for process length was obtained using Neurolucida explorer (MBF Bioscience, Williston, VT, United States). Measurements from individual neurons from each animal were averaged. Total dendritic length was compared among the experimental groups. Branching of the neurons was evaluated using 3D Sholl analysis; for this, the number of dendritic intersections with concentric circles positioned at radial intervals of 20 μm was determined.

### Corticosterone Levels Measurement

For all animals, serum corticosterone levels were measured using a commercially available ELISA kit (Abcam, Cambridge, United Kingdom), according to the manufacturer’s instructions. Blood sampling (tail venipuncture) was performed during the diurnal nadir (N, 8:00–9:00 a.m.) at the end of the stress protocol. Results are expressed as ng of corticosterone *per* ml of serum. Absorbance at 450 nm was determined using a microplate reader and corticosterone concentration (ng/ml) was extrapolated from a standard curve. The coefficient of variation for intra- assay was 5.7% and for inter-assay was 10.2%.

### Estrous Cycle Analysis

In the day of sacrifice, vaginal cytology was performed. Exfoliate cytology was examined under light microscope (Leica DM 4000B, Leica, Wetzlar, Germany) with a 10x objective lens (Plan 63x/0.25PH1) and estrous cycle was determined based on the morphology of the cells present in the smear as previously described (Westwood, 2008).

### Data Analysis

All data are presented as mean ± standard error of the mean (mean ± SEM). GraphPad Prism 6 Software was employed for statistical analysis. Outliers were identified using GraphPad Prism 6. Two-way Analysis of Variance (ANOVA) followed by a Bonferroni *post-hoc* test was used to assess the effects of stress (Control vs. Stress) and duration of stress (Short- vs. Long-term uCMS). The level of significance for all analysis was a set at *p* < 0.05.

## Results

### Unpredictable Chronic Mild Stress Induces a Dysregulation of the Circadian Corticosterone Secretion Pattern in Both Sexes and in the Body Weight of Males

It is known that stress impairs the activity of the hypothalamus-pituitary-adrenal (HPA) axis and results in disrupted secretion of corticosteroids (Pariante and Lightman, 2008; Willner et al., 2013). In this work, we exposed animals of both sexes to a well-established uCMS protocol (Willner, 2005; Bessa et al., 2009a; Mateus-Pinheiro et al., 2013) for either 2 or 6 weeks. To validate the uCMS protocol, we measured corticosterone levels as an indicator of HPA axis function. In basal conditions, control females exhibited higher corticosterone levels than males. At the end of short- and long-term uCMS protocol, basal serum corticosterone levels were higher in both sexes exposed to stress [females: Figure 1B; *F*_(1,24)_ = 10.94, *p* = 0.003; males: Figure 1C; *F*_(1,30)_ = 15.28; *p* = 0.0005], although only statistically significant in the case of the short-term protocol. We also monitored weekly the body weight until the end of the uCMS protocol. In the case of females, short and long uCMS protocols did not significantly affect total body weight [*F*_(1,55)_ = 1.79, *p* = 0.19; Figures 1D,E]. Male rats exposed to uCMS displayed a reduction of body weight [*F*_(1,56)_ = 12.02, *p* = 0.001] after completion of short- (*post-hoc* analysis, *p* = 0.0277) or long- term (*post-hoc* analysis, *p* = 0.0388) uCMS protocols (Figures 1F,G).

### Unpredictable Chronic Mild Stress Induces Anxiety- and Depressive-Like Behavior That Is More Pronounced in Males

**FIGURE 2:**
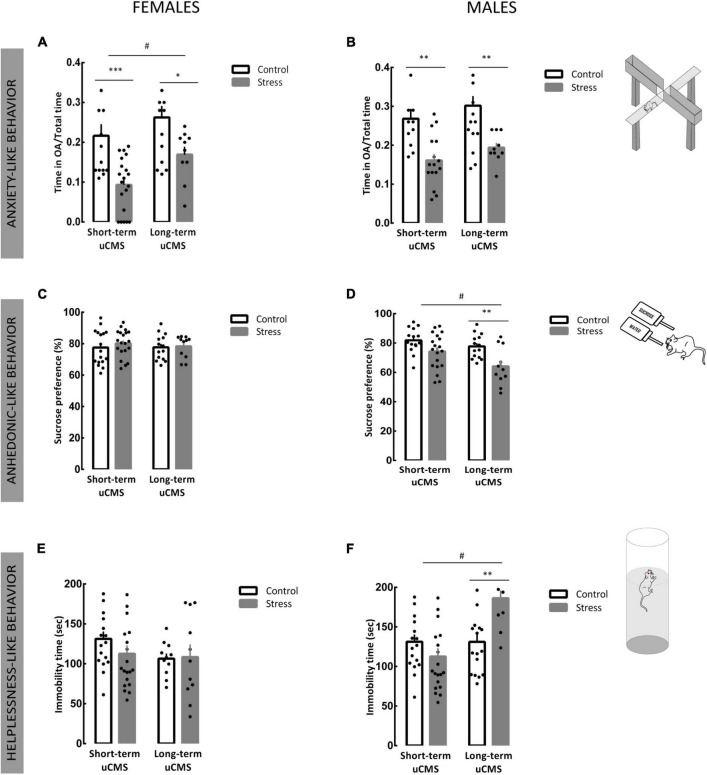
Unpredictable chronic mild stress induces anxiety and depressive-like behavior, an effect more pronounced in males. **(A,B)** Time spent in open arms *per* total time of the elevated plus maze (EPM) test performed to evaluate anxiety-related behavior of females and males. **(C,D)** Anhedonic-like behavior assessed by the preference for sucrose in the sucrose preference test (SPT) in females and males. **(E,F)** Depressive-like behavior assessed by the total time of immobility in the forced swimming test (FST) for females and males. Results are presented as the mean ± SEM of 10–20 animals comparing with control, calculated using a two-way Analysis of Variance (ANOVA) followed by a Bonferroni *post-hoc* test. *p < 0.05, ^**^p < 0.01, and ^***^p < 0.001. ^#^*p* < 0.05 (stress effect).

Unpredictable chronic mild stress induced anxiety-like behavior in females [*F*_(1,54)_ = 19.97, *p* < 0.0001], as demonstrated by the reduced time spent in open arms, after 2 (*post-hoc* analysis, *p* = 0.001) or 6 weeks of uCMS (*post-hoc* analysis, *p* = 0.029; Figure 2A). Like females, we also found a significant effect of stress in males [*F*_(1,50)_ = 23.40, *p* < 0.0001]. Males exposed to a short- or long-term uCMS presented anxiety-like behavior (*post-hoc* analysis, *p* = 0.002; *p* = 0.003, respectively; Figure 2B).

In the SPT, that evaluates anhedonia, no main effect of stress [*F*_(1,58)_ = 0.4701, *p* = 0.4957] or duration of exposure to uCMS [*F*_(1,58)_ = 0.138, *p* = 0.712] was found in females when assessing the percentage of sucrose solution consumed (Figure 2C). In males, we observed a significant stress effect [*F*_(1,55)_ = 14.24, *p* = 0.0004], although only males exposed to a long-term protocol of uCMS showed a decrease in sucrose consumption when compared with controls (*post-hoc* analysis, *p* = 0.004; Figure 2D) with an increase in water consumption (Supplementary Figure 2).

In the FST, behavioral despair was calculated as time of immobility. In females, no differences in immobility were observed (Figure 2E). In males, a main effect of duration of exposure to stress [*F*_(1,61)_ = 12.41, *p* = 0.0008] was found since males exposed to a long-term uCMS showed significantly higher levels of despair behavior, when compared to controls (*post-hoc* analysis, *p* = 0.0019; Figure 2F).

The estrous cycle analysis was performed in females and demonstrated that females were distributed by all phases of the estrous cycle (Supplementary Table 1).

### Unpredictable Chronic Mild Stress Induces Sex-Dependent Morphologic Adaptation of Microglia

Our group described that prenatal stress induces changes in the morphology of microglia (Caetano et al., 2017; Duarte et al., 2019; Gaspar et al., 2021), but the effect of uCMS with different duration and potential sex differences have not been explored. In order to better understand the role of stress in the morphology of microglia, we performed the morphometric analysis of microglia in adult female and male rats in two different brain regions, the dHIP and the NAc. A detailed analysis of microglia, including the number of processes *per* branch order, the total number of branches and the total length of branches was performed.

**FIGURE 3:**
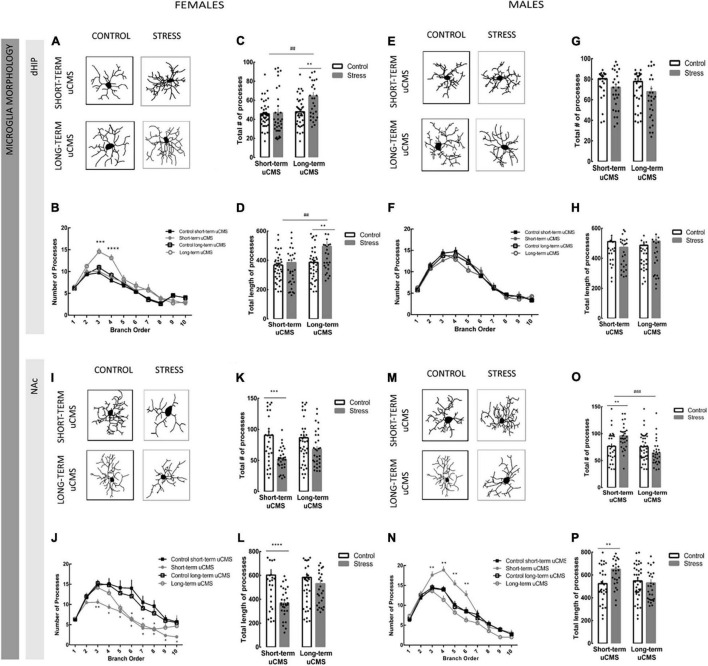
Unpredictable chronic mild stress induces remodeling of microglia, an effect more pronounced in females. Microglial morphometric structure was manually reconstructed in the Neurolucida software based on 3D images of Iba-1 stained microglia. **(A)** Representative microglia cells of the dorsal hippocampus (dHIP) in females. **(B)** Number of processes *per* branch of microglia of the dHIP in females. **(C,D)** Total number and length of microglia cells of the dHIP in females. **(E)** Representative microglia cells of the dHIP in males. **(F)** Number of processes *per* branch of microglia of the dHIP in males. **(G,H)** Total number and length of microglia cells of the dHIP in males. **(I)** Representative microglia cells from of the *nucleus accumbens* (NAc) in females. **(J)** Number of processes *per* branch of microglia of the NAc in females. **(K,L)** Total number and length of microglia cells of the NAc in females. **(M)** Representative microglia cells of the NAc in males of the NAc. **(N)** Number of processes per branch of microglia of the NAc in males. **(O,P)** Total number and length of microglia cells of the NAc in females. Results are presented as the mean ± SEM of 40–50 cells from 4 to 5 animals; comparing with control, calculated using a two-way ANOVA followed by a Bonferroni *post-hoc* test. *p < 0.05, ^**^p < 0.01, ***p < 0.001, and ****p < 0.0001. ^##^*p* < 0.01 and ^###^*p* < 0.001 (stress effect).

In the dHIP, short-term uCMS did not induce alterations in microglia morphology in females (Figures 3A–D and Supplementary Table 2). Conversely, long-term exposure to uCMS induced a hypertrophy of microglia in the dHIP in females, when compared to control animals, either in the total number of processes (*post-hoc* analysis, *p* = 0.002) and in total length [*F*_(1,140)_ = 3.08, *p* = 0.005; Figures 3C,D]. In males, we did not observe any effect of stress (both short or long) in the morphology of microglia either in terms of total number or length (Figures 3E–H and Supplementary Table 2).

In the NAc, we observed opposite differences between sexes. Short-term uCMS in females induced a general decrease in the total number of processes [*F*_(1,126)_ = 19.47, *p* < 0.0001; Figures 3I,K] and in the length [*F*_(1,126)_ = 14.23, *p* = 0.0002; Figures 3I,L] (atrophy). Long-term uCMS induced also a decrease of microglia morphology, but only in the number of processes *per* branch order (Figure 3J and Supplementary Table 3). On the other hand, males exposed to short-term uCMS presented an increase in the total number of processes [*F*_(1,123)_ = 0.69; *p* = 0.0088] and in the length (hypertrophy) [*F*_(1,123)_ = 3.49, *p* = 0.0069] of NAc microglia, but long-term uCMS did not induce alterations in microglia morphology in males (Figures 3M–P and Supplementary Table 3).

When we compared microglia morphology under physiological conditions in both regions, we observed that microglia cells of females in the NAc exhibited a more complex morphology compared with dHIP. No differences between dHIP and NAc were observed in males microglia (Supplementary Figures 3A,B and Supplementary Table 4).

### Unpredictable Chronic Stress Induces Contrasting Patterns of Neuronal Dendritic Remodeling in the Dorsal Hippocampus and *Nucleus Accumbens* in Males

Neuronal morphology was assessed by three-dimensional morphometric analysis of Golgi impregnated granule neurons in the DG of dHIP and spiny medium neurons in NAc.

**FIGURE 4:**
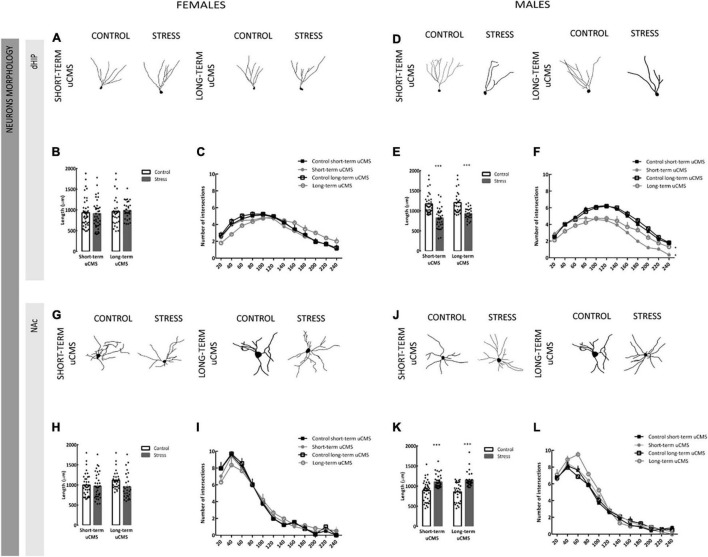
Unpredictable chronic mild stress induces remodeling of neurons only in males. **(A)** Representative manual reconstruction of Golgi-impregned granule neurons of the dorsal hippocampus (dHIP) in females. **(B,C)** Total dendritic length and sholl analysis of dendritic distribution of neurons in the dentate gyrus of the dHIP in females. **(D)** Representative manual reconstruction of Golgi-impregned granule neurons of the dHIP in males. **(E,F)** Total dendritic length and sholl analysis of dendritic distribution of neurons in the dentate gyrus of the dHIP in males. **(G)** Representative manual reconstruction of Golgi-impregned medium spiny neurons of the *nucleus accumbens* (NAc) in females. **(H,I)** Total dendritic length and sholl analysis of dendritic distribution of neurons in the NAc in females. **(J)** Representative manual reconstruction of Golgi-impregned medium spiny neurons of the NAc in males. **(K,L)** Total dendritic length and sholl analysis of dendritic distribution of neurons of the NAc in males. Results are presented as the mean ± SEM of 30–40 cells from 3 to 4 animals; comparing with control, calculated using a two-way Analysis of Variance (ANOVA) followed by a Bonferroni *post-hoc* test. *p < 0.05, ^**^p < 0.01, and ^***^p < 0.001.

Unpredictable chronic mild stress revealed no significant effect in the morphology or in the Sholl analysis of neurons of the dHIP in females (Figures 4A–C). In males, exposure to stress induced an atrophy of granule neurons of the dHIP, with a significant decrease in their total dendritic length [*F*_(1,10)_ = 59.75, *p* < 0.00001] as compared with neurons of control animals (Figures 4D–F). Both short- (*post-hoc* analysis, *p* = 0.0003) and long-term of CMS (*post-hoc* analysis, *p* = 0.0008) significantly decreased total dendritic length in granule neurons of the dHIP (Figure 4E). In Sholl analysis we also observed an effect of stress: males presented a less complex morphology when compared with controls [*F*_(3,120)_ = 53.39, *p* < 0.00001; Figure 4F].

We next analyzed the morphological effects of stress in NAc neurons. In females we did not observe any effect of stress in the morphology or in the Sholl analysis of spiny medium neurons (Figures 4G–I). Contrarily to what we observed in the dHIP, uCMS induced a hypertrophy in the NAc medium spiny neurons of males, which displayed a significant increase in dendritic length [*F*_(1,10)_ = 79.65, *p* < 0.00001; Figures 4J,K]. Both short- (*post-hoc* analysis, *p* = 0.0003) and long-term CMS (*post-hoc* analysis, *p* = 0.0001) significantly increased total dendritic length of medium spiny neurons (Figure 4K). Sholl analysis revealed more complex medium spiny neurons in males exposed to long-term uCMS compared to controls [*F*_(3,112)_ = 3.122, *p* = 0.028; Figure 4L].

## Discussion

The present study explored how short- and long-term uCMS at adulthood alters behavior in males and females and identified changes in the morphology of microglia and neurons of the dHIP and NAc. This issue deserves particular investigation, considering that the process of morphologic remodeling of neurons and microglia is usually accompanied by functional changes with behavioral expression.

The uCMS model is one of the most widely used rodent models to produce behavioral deficits and neuroplastic changes with strong face validity to human depression, that include not only anhedonia, but also anxiety and cognitive impairments in spatial memory and object recognition tasks (Willner et al., 1987; Willner, 1997, 2005; Bessa et al., 2013). However, the differential risk for anxiety and depressive-like behavior between sexes considering a short- (2 weeks) and long-term (6 weeks) uCMS protocol is still not fully elucidated, in particular in what concerns to the characterization of cellular (neurons and microglia) plasticity in an attempt to find a correlation pattern. Considering the marked differences in the prevalence of depression in men and women (Marcus et al., 2005), there has been a considerable interest in sex specificities in anxiety- and depression-like symptoms expressed in animals exposed to stress. Nevertheless, sex differences in the risk and resilience to stress are complex and vary according to the characteristics of the stressor, such as timing, type and severity (Hodes and Epperson, 2019). The basis for these differences is unknown, in part because much of the work in the field is performed mostly in male rodents (Klein et al., 2015), perhaps due to the challenges associated with carrying out experiments influenced by fluctuating gonadal hormones in females (O’Connor and Barrett, 2014).

First, our results showed that body weight is affected (reduced) in males, but not in female rats after short- or long-term uCMS protocols. Although consistent with several studies, showing that chronic stress has a higher impact in reducing male weight gain (Konkle et al., 2003; Mateus-Pinheiro et al., 2013; Patricio et al., 2015), it is important to consider the influence of conditions, such as the type and the intensity of stressor, as well as the age of stress onset. For instance, chronic stress in late adolescent female animals reduces body weight gain (Wulsin et al., 2016).

Assessment of corticosterone levels as an index of the stress response revealed higher levels in both sexes exposed to uCMS comparing to control animals. It is important to note that females have higher basal concentrations of corticosterone and secrete higher levels after stress exposure, as previously described by other authors (Kitay, 1961; Goel et al., 2014; Oyola and Handa, 2017).

In behavioral tests, we showed that male rats are more affected than females by these protocols of stress. Both sexes exhibited anxiety-like behavior in response to stress, but only male rats presented anhedonia and despair-like behavior, cardinal symptoms of depression.

Unpredictable chronic mild stress-induced anxiety-like behavior in both sexes is consistent with other studies showing that animals exposed to chronic stress spent less time in the open arms in the EPM test (Kompagne et al., 2008; Yue et al., 2017; Wang et al., 2018).

Furthermore, 6 weeks of uCMS lead to anhedonia and helplessness/despair behaviors in male animals, core symptoms of depression that have been also described as characteristics of stress-related conditions (D’Aquila et al., 1994; Willner et al., 1996; Bekris et al., 2005; Bessa et al., 2009a; Patricio et al., 2015). Stress-induced differences in sucrose consumption between males and females were somehow expected due to sex differences in taste and/or ingestion responses (Clarke and Ossenkopp, 1998; Curtis et al., 2004) or in reactivity to reward (Michaels and Holtzman, 2007). Indeed, other studies support the present observation that stress-induced alterations in sucrose consumption are differently expressed between male and female animals (Dalla et al., 2005, 2008; Pitychoutis et al., 2009). Regarding despair behavior (here assessed by the FST), our data, although in line with other studies [showing that females exposed to CMS cope better and present increased active behavior in the FST, whereas males are more vulnerable (Bielajew et al., 2003; Dalla et al., 2005)] are particularly intriguing because in humans, depression is more prominent in females (Frank et al., 1988; Marcus et al., 2005; Wittchen et al., 2011).

In this study we explored the effect of stress on microglia morphology in the dHIP and NAc, two key brain regions in the control of depressive-like behavior (Di Chiara et al., 1999; Nestler, 2001; Nestler and Carlezon, 2006; Bessa et al., 2013; Alves et al., 2018). Microglia are diverse in shape and function and may present phenotypic differences according to the brain region analyzed (Caetano et al., 2017; De Biase et al., 2017; De Biase and Bonci, 2019; Duarte et al., 2019; Gaspar et al., 2021) and determined by sex (Lenz et al., 2013; Caetano et al., 2017; Villapol et al., 2017; Gaspar et al., 2021). All these variables may contribute to adapted functional responses to different insults (Schwarz et al., 2012; Villapol et al., 2017; Guneykaya et al., 2018; Perkins et al., 2018; Villa et al., 2018). Thus, it is not surprising that chronic stress elicits brain region- and sex-specific alterations in microglial phenotypes that likely contribute to divergent neurobiological and behavioral responses (Hinwood et al., 2013; Kreisel et al., 2014; Milior et al., 2016; Franklin et al., 2018). In the dHIP, microglia from males are not affected by chronic stress (shorter or longer periods of exposure), while females, although requiring a longer period of exposure to stress, present hypertrophied microglia (more and longer cellular processes). In line with these results, our group described that prenatal stress exposure induces a hypertrophy of microglia in females with no differences in males (Gaspar et al., 2021). These findings are consistent with other study reporting the absence of changes in the morphology of microglia in males in the HIP following chronic stress (Lehmann et al., 2016). In the case of NAc, microglia from both sexes is affected by stress, but changes observed after 2 weeks of stress are apparently transient and no longer observed after 6 weeks of stress exposure. To our knowledge, our group described for the first time alterations in microglia morphology in the NAc after stress exposure. Recently we showed that prenatal exposure to stress induced also sex-specific alterations in microglia (atrophy in females and hypertrophy in males) (Gaspar et al., 2021). It is becoming evident that microglia morphology is robustly and differently affected by stress in different brain regions. For example, 21 days of restraint stress increased the complexity of microglia in males, enhancing ramifications in the PFC (Hinwood et al., 2013). Studies from our team have shown that prenatal stress triggers long-lasting sex differences in microglia morphology in the mPFC, dHIP, and NAc (Caetano et al., 2017; Duarte et al., 2019; Gaspar et al., 2021). Given that microglia present sexual dimorphic features, namely density, function, and morphology in several brain regions (Bilbo et al., 2012; Schwarz and Bilbo, 2012; Schwarz et al., 2012; Caetano et al., 2017; Duarte et al., 2019; Gaspar et al., 2021), some of which conserved among species (Simoes-Henriques et al., 2019), sex differences after stress are not surprising.

The morphologic adaptation of neurons to stress has been also studied by several authors. In general, it is accepted that stress induces an atrophy of neurons in the HIP (Watanabe et al., 1992; Magarinos and McEwen, 1995; McLaughlin et al., 2007; Bessa et al., 2009a; Morais et al., 2014; Patricio et al., 2015) and a hypertrophy in the NAc (Bessa et al., 2013; Melo et al., 2015). One of the main goals of this work was to analyze behavior and, in parallel, microglia and neurons morphology. Interestingly, the morphometric analysis of neurons in the dHIP revealed that these cells are morphologically not responsive to stress in the case of females, but males present an atrophic pattern after 2 weeks of stress, an effect that persists until 6 weeks of stress. In the NAc, only males present changes (conversely to the dHIP, a hypertrophy was observed), which are observable after a short protocol of stress and persist after longer periods of exposure.

In summary, neuronal changes in this brain region seem to be exclusive to males and opposite between dHIP and NAc. In line with our results, some studies (only performed in males) demonstrated that chronic stress caused an atrophy of neurons in the DG of HIP (Bessa et al., 2009b; Morais et al., 2014; Patricio et al., 2015) and in the mPFC (Radley et al., 2005; Shansky et al., 2009; Melo et al., 2015). Interestingly, chronic adult stress triggered a hypertrophy of medium spiny neurons in the NAc, that was associated with a depressive-like phenotype (Bessa et al., 2013; Melo et al., 2015). Thus, the NAc neuronal hypertrophy that we observed in this study can contribute for the depressive-like phenotype that is observed in males. In this framework, the lack of changes in females is in agreement with the absence of a phenotype in the SPT and FST.

## Conclusion

**FIGURE 5:**
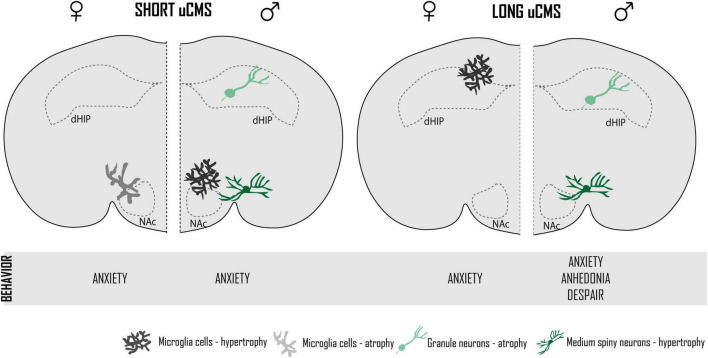
Unpredictable chronic mild stress alters the behavior and the morphology of microglia and neurons in a brain region- and sex-specific manner.

The present results show that chronic stress significantly alters the behavior and the morphology of microglia and neurons in a brain region- and sex-specific manner: males are more affected by stress, presenting anxiety- and depression-like behaviors, hypertrophy of microglia, and dendritic hypertrophy in the NAc. Females present anxiety-like behavior, but no depression-like behavior, with remodeling of microglia in dHIP (hypertrophy) and NAc (atrophy) (Figure 5). Globally, our results show that the morphology of neurons is not affected by chronic stress in females and this morphologic stability is accompanied by a process of microglia remodeling. In the case of males, neurons are affected in both regions, but microglia seem to be only and transiently affected in the NAc. This study led us to question if microglia plasticity is related with the morphologic stability of neurons observed in females, eventually underlying stress resilience, a hypothesis that deserves further investigation.

## Data Availability Statement

The original contributions presented in the study are included in the article/Supplementary Material, further inquiries can be directed to the corresponding authors.

## Ethics Statement

The animal study was reviewed and approved by EU-Directive 2010/63/EU and the Portuguese National Authority for animal experimentation, Direção-Geral de Animal e Veterinária (DGAV). All protocols were approved by the Ethics Committee of the Life and Health Sciences Research Institute and by DGAV (#19074).

## Author Contributions

RG designed the experiments with CS-C, AR, and CG, performed the experiments, and wrote the manuscript. CS-C, AR, and BC helped to perform behavioral experiments. CG and AR supervised RG, contributed to the design of the experiments, and revised the manuscript. All authors discussed the results, contributed to the article, and approved the final submitted version.

## Conflict of Interest

The authors declare that the research was conducted in the absence of any commercial or financial relationships that could be construed as a potential conflict of interest.

## Publisher’s Note

All claims expressed in this article are solely those of the authors and do not necessarily represent those of their affiliated organizations, or those of the publisher, the editors and the reviewers. Any product that may be evaluated in this article, or claim that may be made by its manufacturer, is not guaranteed or endorsed by the publisher.
