# Supplementary material for: The Duration of Stress Determines Sex Specificities in the Vulnerability to Depression and in the Morphologic Remodeling of Neurons and Microglia
Source: Front Behav Neurosci. 2022 Mar 7;16:834821. doi: 10.3389/fnbeh.2022.834821 (PMC8940280; doi:10.3389/fnbeh.2022.834821)
Supplement: Supplementary file 1 [file Image_1.pdf]

Week 1	Mon	Tue	Wed	Thu	Fri	Sat	Sun
9h - 10h	Confinement	Cage replacement	Inacc. Food	Overcrowding	Cage replacement		
10h - 11h		Empty Bottle	Confinement		Confinement		
11h - 12h	Strobe Lights	Startle Noise					
12h - 13h			Tilted Cage		Tilted Cage		
13h - 14h				Cage Switch			
14h - 15h	Tilted Cage	Overcrowding			Strobe Lights		
15h - 16h				Startle Noise			
16h - 17h							
17h - 18h							
O/N	Water Dep. + wet bed	Food deprivation+ ON Illumination	Strobe lights	Wet Bed	Rev. Light + Tilted Cage		

Week 2	Mon	Tue	Wed	Thu	Fri	Sat	Sun
9h - 10h	Confinement	Cage replacement	Empty Bottle	Cage Switch	Cage replacement		
10h - 11h		Inacc. Food	Overcrowding		Startle Noise		
11h - 12h	Startle Noise	Tilted Cage					
12h - 13h					Confinement		
13h - 14h				Tilted Cage			
14h - 15h	Overcrowding		Confinement		Overcrowding		
15h - 16h							
16h - 17h		Strobe lights (LF)	Startle Noise				
17h - 18h							
O/N	Food Dep. + wet bed	Water deprivation+ ON Illumination	Strobe lights	Wet Bed	Inv. Light + Tilted Cage		

Week 3	Mon	Tue	Wed	Thu	Fri	Sat	Sun
9h - 10h	Tilted Cage	Cage replacement	Inacc. Food	Overcrowding	CageReplac.		
10h - 11h		Empty Bottle	Confinement		Confinement		
11h - 12h		Startle Noise			Strobe Lights		
12h - 13h			Tilted Cage				
13h - 14h	Confinement			Cage Switch			
14h - 15h	Strobe Lights	Overcrowding			Tilted Cage		
15h - 16h				Startle Noise			
16h - 17h							
17h - 18h							
O/N	Water Dep. + wet bed	Food Dep.+O/N illumination	Strobe Lights	Wet Bed	Rev. Light + Tilted Cage		

Week 4	Mon	Tue	Wed	Thu	Fri	Sat	Sun
9h - 10h	Confinement	Cage replacement	Empty Bottle	Cage Switch	CageReplac.		
10h - 11h		Inacc. Food	Overcrowding		Startle Noise		
11h - 12h	Startle Noise	Strobe Lights					
12h - 13h					Confinement		
13h - 14h		Tilted Cage	Confinement				
14h - 15h	Overcrowding		Startle Noise	Tilted Cage	Overcrowding		
15h - 16h							
16h - 17h							
17h - 18h							
O/N	Food Dep. + wet bed	water Dep. + O/N Illum.	Strobe Lights	Wet Bed	Rev. Light		

Week 5	Mon	Tue	Wed	Thu	Fri	Sat	Sun
9h - 10h	Cage Switch	Cage replacement	Empty Bottle	Overcrowding	CageReplac.		
10h - 11h		Inacc. Food	Strobe Lights		Confinement		
11h - 12h		Tilted cage			Startle Noise		
12h - 13h							
13h - 14h	Strobe Lights		Startle Noise	Tilted Cage			
14h - 15h					Strobe Lights		
15h - 16h		Overcrowding		Cage Switch			
16h - 17h	Confinement		Confinement				
17h - 18h							
O/N	Food Dep. + wet bed	water Dep. + O/N Illum.	Strobe lights	Wet Bed	Rev. Light + Tilted Cage		

Week 6	Mon	Tue	Wed	Thu	Fri	Sat	Sun
9h - 10h	Tilted cage	Inacc. Food	Empty Bottle	Overcrowding	CageReplac.		
10h - 11h		1h Food Intake	Strobe Lights		Strobe Lights		
11h - 12h		Overcrowding					
12h - 13h							
13h - 14h	Strobe Lights		Startle Noise	Startle Noise	Tilted Cage		
14h - 15h		Tilted cage					
15h - 16h							
16h - 17h	Confinement		Confinement		Confinement		
17h - 18h							
O/N	Food Dep.	water Dep. + O/N Illum.	Strobe lights	Wet Bed	Rev. Light + Tilted Cage		

Supplementary Figure 1 – Schedule of unpredictable chronic mild stressors administered over a 7-day period and repeated for 2 or 6 weeks.

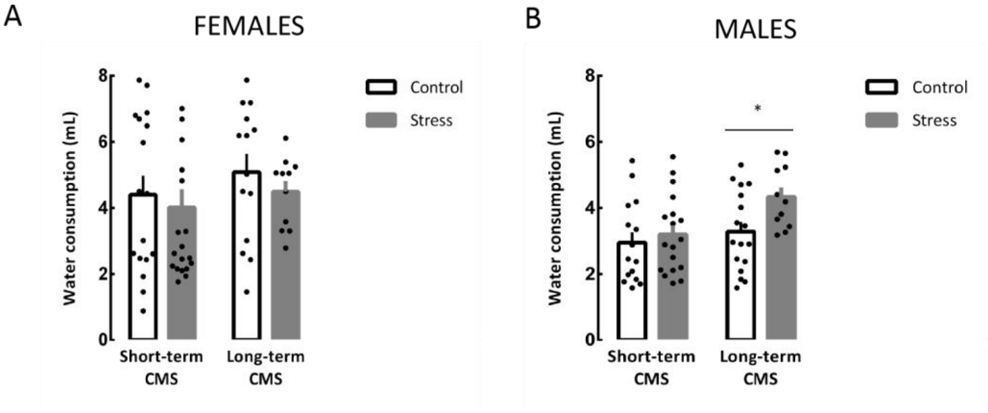

Supplementary Figure 2 – Water consumption in Sucrose Preference Test (SPT) in females and males. Results are presented as the mean ± SEM of 10-20 animals comparing with control, calculated using a two-way ANOVA followed by a Bonferroni post hoc test. *p<0.05.

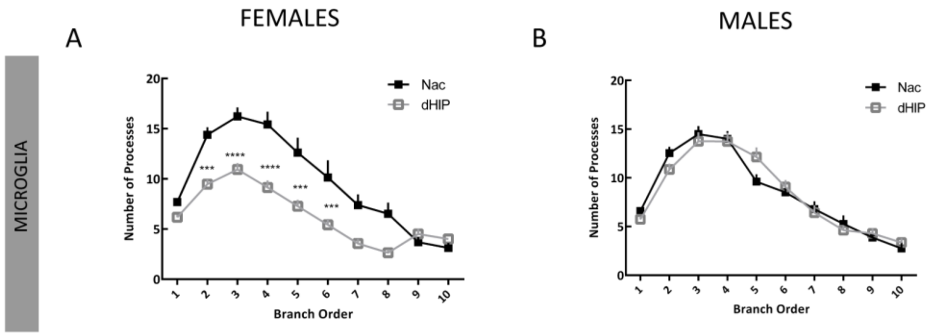

Supplementary Figure 3 – Microglia morphology is more complex in the Nac than in the dHIP in females. (A, B) Comparison of the number of microglial processes from dHIP and NAc of control animals according to the respective branch order in females and males. Results are presented as the mean ± SEM of 30-40 cells from 3-4 animals, calculated using a one-way ANOVA followed by a Bonferroni post hoc test, ***$p<0.05$.
