# Supplementary material for: The Duration of Stress Determines Sex Specificities in the Vulnerability to Depression and in the Morphologic Remodeling of Neurons and Microglia
Source: Front Behav Neurosci. 2022 Mar 7;16:834821. doi: 10.3389/fnbeh.2022.834821 (PMC8940280; doi:10.3389/fnbeh.2022.834821)
Supplement: Supplementary file 2 [file Table_1.pdf]

Supplementary Table 1 – Female rats estrous cycle determined in the day of sacrificed.

		Proestrus	Estrus	Metestrus	Diestrus
**SHORT- TERM**	Control	5	3	5	0
	Stress	4	3	4	1
**LONG-TERM**	Control	4	2	8	1
	Stress	6	2	7	0
