# Supplementary material for: The Duration of Stress Determines Sex Specificities in the Vulnerability to Depression and in the Morphologic Remodeling of Neurons and Microglia
Source: Front Behav Neurosci. 2022 Mar 7;16:834821. doi: 10.3389/fnbeh.2022.834821 (PMC8940280; doi:10.3389/fnbeh.2022.834821)
Supplement: Supplementary file 3 [file Table_2.pdf]

Supplementary Table 2 - Morphometric data and statistics for microglia morphology from dHIP of females and males at PND exposed to short- and long-term uCMS: Number and length of ramifications

MORPHOMETRIC DATA and STATISTICS **dHIP – FEMALES**

NUMBER of PROCESSES

ORDER	Control Short-term uCMS	Short-term uCMS	Control Long-term uCMS	Long-term uCMS
1	6 ± 0.2 (n=40)	6 ± 0.3 (n=34)	6 ± 0.3 (n=40)	6 ± 0.3 (n=30)
2	9 ± 0.4 (n=40)	10 ± 0.5 (n=34)	10 ± 0.5 (n=40)	11 ± 0.5 (n=30)
3	10 ± 0.5 (n=40)	10 ± 0.7 (n=34)	11 ± 0.6 (n=40)	15 ± 0.8 (n=30) ***
4	8 ± 0.6 (n=40)	9 ± 1.0 (n=33)	9 ± 0.7 (n=40)	13 ± 0.9 (n=30)****
5	7 ± 0.6 (n=32)	8 ± 1.1 (n=25)	7 ± 0.6 (n=26)	8 ± 1.0 (n=30)
6	5 ± 0.5 (n=28)	6 ± 1.0 (n=17)	5 ± 0.6 (n=26)	7 ± 1.0 (n=25)
7	4 ± 0.5 (n=19)	6 ± 1.0 (n=11)	4 ± 0.5 (n=16)	6 ± 1.0 (n=16)
8	3 ± 0.4 (n=12)	4 ± 0.6 (n=9)	3 ± 0.3 (n=11)	4 ± 0.7 (n=11)
9	5 ± 0.5 (n=4)	3 ± 0.9 (n=5)	5 ± 0.5 (n=4)	3 ± 0.5 (n=5)
10	4 ± 0.0 (n=1)	3 ± 0.7 (n=3)	4 ± 0.0 (n=1)	3 ± 1.0 (n=2)

MORPHOMETRIC DATA and STATISTICS **dHIP – MALES**

NUMBER of PROCESSES

ORDER	Control Short-term uCMS	Short-term uCMS	Control Long-term uCMS	Long-term uCMS
1	6 ± 0.3 (n=25)	6 ± 0.4 (n=30)	6 ± 0.3 (n=34)	6 ± 0.4 (n=30)
2	11 ± 0.6 (n=25)	11 ± 0.6 (n=30)	11 ± 0.6 (n=34)	12 ± 0.7 (n=30)
3	14 ± 0.9 (n=25)	13 ± 0.7 (n=30)	14 ± 0.9 (n=34)	14 ± 1.1 (n=30)
4	15 ± 1.0 (n=25)	14 ± 0,8 (n=30)	14 ± 0.9 (n=34)	13 ± 1.1 (n=29)
5	13 ± 1.2 (n=25)	12 ± 1.0 (n=30)	12 ± 1.0 (n=34)	10 ± 1.0 (n=29)
6	9 ± 0.8 (n=24)	10 ± 0,9 (n=27)	9 ± 0.7 (n=33)	9 ± 1.2 (n=20)
7	6 ± 0.8 (n=23)	6 ± 0,7 (n=25)	6 ± 0.8 (n=31)	6 ± 1.1 (n=19)
8	5 ± 0.5 (n=21)	4 ± 0.6 (n=17)	5 ± 0.5 (n=27)	4 ± 0.7 (n=14)
9	4 ± 0.5 (n=13)	5 ± 0.8 (n=7)	4 ± 0.5 (n=17)	4 ± 0.9 (n=7)
10	3 ± 0.5 (n=9)	3 ± 0.9 (n=5)	3 ± 0.4 (n=11)	4 ± 0.3 (n=4)
