# Supplementary material for: The Duration of Stress Determines Sex Specificities in the Vulnerability to Depression and in the Morphologic Remodeling of Neurons and Microglia
Source: Front Behav Neurosci. 2022 Mar 7;16:834821. doi: 10.3389/fnbeh.2022.834821 (PMC8940280; doi:10.3389/fnbeh.2022.834821)
Supplement: Supplementary file 4 [file Table_3.pdf]

Supplementary Table 3 - Morphometric data and statistics for microglia morphology from NAc of females and males at PND exposed to short- and long-term uCMS: Number and length of ramifications

MORPHOMETRIC DATA and STATISTICS				**NAc– FEMALES**
**NUMBER** of PROCESSES				
**ORDER**	**Control Short-term CMS**	**Short-term CMS**	**Control Long-term CMS**	**Long-term CMS**
1	6 ± 0.4 (n=30)	7 ± 0.4 (n=30)	6 ± 0.4 (n=40)	6 ± 0.4 (n=30)
2	12 ± 0.8 (n=30)	10 ± 0.7 (n=30)	12 ± 0.7 (n=40)	12 ± 0.8 (n=30)
3	15 ± 1.0 (n=30)	11 ± 0.7 (n=30)*	15 ± 0.9 (n=40)	14 ± 1.0 (n=30)
4	15 ± 1.4 (n=30)	9 ± 0.8 (n=30)***	15 ± 1.1 (n=40)	13 ± 1.1 (n=30)
5	14 ± 1.8 (n=28)	8 ± 0.8 (n=27)***	13 ± 1.4 (n=38)	9 ± 0.8 (n=30)
6	14 ± 2.1 (n=25)	6 ± 0.8 (n=20)****	12 ± 1.7 (n=34)	6 ± 0.8 (n=29)*
7	11 ± 1.6 (n=22)	4 ± 0.9 (n=14)**	9 ± 1.3 (n=30)	5 ± 0.7 (n=24)***
8	10 ± 1.5 (n=18)	4 ± 0.7 (n=9)	8 ± 1.3 (n=24)	4 ± 0.7 (n=15)*
9	6 ± 0.9 (n=16)	2 ± 0.3 (n=6)	6 ± 0.9 (n=18)	4 ± 1.1 (n=7)
10	6 ± 0.9 (n=11)	2 ± 0.0 (n=1)	6 ± 0.8 (n=12)	5 ± 2.7 (n=3)

MORPHOMETRIC DATA and STATISTICS				**NAc– MALES**
**NUMBER** of PROCESSES				
**ORDER**	**Control Short-term CMS**	**Short-term CMS**	**Control Long-term CMS**	**Long-term CMS**
1	6 ± 0.5 (n=29)	7 ± 0.5 (n=30)	7 ± 0.4 (n=39)	7 ± 0.4 (n=29)
2	12 ± 0.7 (n=29)	13 ± 0.8 (n=30)	13 ± 0.6 (n=39)	13 ± 0.8 (n=29)
3	14 ± 1.0 (n=29)	18 ± 0.9 (n=30)*	14 ± 0.8 (n=39)	14 ± 1.0 (n=29)
4	14 ± 0.9 (n=29)	19 ± 1.0 (n=30)***	14 ± 0.8 (n=39)	11 ± 1.1 (n=29)
5	10 ± 0.9 (n=29)	15 ± 1.0 (n=30)****	10 ± 0.8 (n=39)	8 ± 0.9 (n=29)
6	8 ± 1.1 (n=27)	13 ± 1.1 (n=29)**	9 ± 0.9 (n=36)	6 ± 0.9 (n=28)
7	8 ± 1.0 (n=24)	8 ± 0.9 (n=25)	7 ± 0.8 (n=33)	6 ± 1.3 (n=16)
8	5 ± 0.9 (n=19)	4 ± 0.7 (n=21)	5 ± 0.9 (n=22)	4 ± 0.6 (n=11)
9	4 ± 0.7 (n=13)	4 ± 0.6 (n=13)	4 ± 0.7 (n=15)	2 ± 0.0 (n=5)
10	3 ± 0.6 (n=8)	3 ± 0.7 (n=8)	3 ± 0.6 (n=8)	2 ± 0.0 (n=2)
