# Supplementary material for: The Duration of Stress Determines Sex Specificities in the Vulnerability to Depression and in the Morphologic Remodeling of Neurons and Microglia
Source: Front Behav Neurosci. 2022 Mar 7;16:834821. doi: 10.3389/fnbeh.2022.834821 (PMC8940280; doi:10.3389/fnbeh.2022.834821)
Supplement: Supplementary file 5 [file Table_4.pdf]

Supplementary Table 4 - Morphometric data and statistics for microglia morphology from dHIP and NAc of females and males at PND 90 in physiological conditions: Number and length of ramifications

MORPHOMETRIC DATA and STATISTICS **FEMALES**

NUMBER of PROCESSES

ORDER	dHIP	NAc
1	6 ± 0.2 (n=40)	6 ± 0.4 (n=30)
2	9 ± 0.4 (n=40)	12 ± 0.8 (n=30)
3	10 ± 0.5 (n=40)	15 ± 1.0 (n=30) ****
4	8 ± 0.6 (n=40)	15 ± 1.4 (n=30) ****
5	7 ± 0.6 (n=32)	14 ± 1.8 (n=28) ****
6	5 ± 0.5 (n=28)	14 ± 2.1 (n=25) ***
7	4 ± 0.5 (n=19)	11 ± 1.6 (n=22)
8	3 ± 0.4 (n=12)	10 ± 1.5 (n=18)
9	5 ± 0.5 (n=4)	6 ± 0.9 (n=16)
10	4 ± 0.0 (n=1)	6 ± 0.9 (n=11)

MORPHOMETRIC DATA and STATISTICS **MALES**

NUMBER of PROCESSES

ORDER	dHIP	NAc
1	6 ± 0.3 (n=25)	6 ± 0.5 (n=29)
2	11 ± 0.6 (n=25)	12 ± 0.7 (n=29)
3	14 ± 0.9 (n=25)	14 ± 1.0 (n=29)
4	15 ± 1.0 (n=25)	14 ± 0.9 (n=29)
5	13 ± 1.2 (n=25)	10 ± 0.9 (n=29)
6	9 ± 0.8 (n=24)	8 ± 1.1 (n=27)
7	6 ± 0.8 (n=23)	8 ± 1.0 (n=24)
8	5 ± 0.5 (n=21)	5 ± 0.9 (n=19)
9	4 ± 0.5 (n=13)	4 ± 0.7 (n=13)
10	3 ± 0.5 (n=9)	3 ± 0.6 (n=8)
