# Supplementary material for: The Duration of Stress Determines Sex Specificities in the Vulnerability to Depression and in the Morphologic Remodeling of Neurons and Microglia
Source: Front Behav Neurosci. 2022 Mar 7;16:834821. doi: 10.3389/fnbeh.2022.834821 (PMC8940280; doi:10.3389/fnbeh.2022.834821)
Supplement: Supplementary file 6 [file Table_5.pdf]

Supplementary Table 5 – Number of animals in each behavioral test.

	FEMALES			MALES		
	Elevated Plus Maze	Sucrose Preference Test	Forced Swimming Test	Elevated Plus Maze	Sucrose Preference Test	Forced Swimming Test
Control (short-term uCMS)	14	17	17	11	15	17
Short-term uCMS	20	20	20	16	19	20
Control (long-term uCMS)	14	14	11	17	14	17
Long-term uCMS	10	11	11	10	11	11
